# Hot and cold executive functions in the brain: A prefrontal-cingular network

**DOI:** 10.1177/23982128211007769

**Published:** 2021-04-23

**Authors:** Mohammad Ali Salehinejad, Elham Ghanavati, Md Harun Ar Rashid, Michael A. Nitsche

**Affiliations:** 1 Department of Psychology and Neurosciences, Leibniz Research Centre for Working Environment and Human Factors, Dortmund, Germany; 2 Department of Neuropsychology, Institute of Cognitive Neuroscience, Faculty of Psychology, Ruhr University Bochum, Bochum, Germany; 3 Department of Neurology, University Medical Hospital Bergmannsheil, Bochum, Germany

**Keywords:** Executive functions, hot–cold cognition, prefrontal cortex, anterior cingulate cortex, posterior cingulate cortex, non-invasive brain stimulation, tDCS, TMS, neuroimaging, fMRI

## Abstract

Executive functions, or cognitive control, are higher-order cognitive functions needed for adaptive goal-directed behaviours and are significantly impaired in majority of neuropsychiatric disorders. Different models and approaches are proposed for describing how executive functions are functionally organised in the brain. One popular and recently proposed organising principle of executive functions is the distinction between *hot* (i.e. reward or affective-related) versus *cold* (i.e. purely cognitive) domains of executive functions. The prefrontal cortex is traditionally linked to executive functions, but on the other hand, anterior and posterior cingulate cortices are hugely involved in executive functions as well. In this review, we first define executive functions, their domains, and the appropriate methods for studying them. Second, we discuss how *hot* and *cold* executive functions are linked to different areas of the prefrontal cortex. Next, we discuss the association of *hot* versus *cold* executive functions with the cingulate cortex, focusing on the anterior and posterior compartments. Finally, we propose a functional model for *hot* and *cold* executive function organisation in the brain with a specific focus on the *fronto-cingular* network. We also discuss clinical implications of *hot* versus *cold* cognition in major neuropsychiatric disorders (depression, schizophrenia, anxiety disorders, substance use disorder, attention-deficit hyperactivity disorder, and autism) and attempt to characterise their profile according to the functional dominance or manifest of *hot–cold* cognition. Our model proposes that the lateral prefrontal cortex along with the dorsal anterior cingulate cortex are more relevant for *cold* executive functions, while the medial–orbital prefrontal cortex along with the ventral anterior cingulate cortex, and the posterior cingulate cortex are more closely involved in *hot* executive functions. This functional distinction, however, is not absolute and depends on several factors including task features, context, and the extent to which the measured function relies on cognition and emotion or both.

## Introduction

### Executive functions and their domains

Executive functions (EFs), also called cognitive control, refer to a family to top-down cognitive processes required for goal-directed behaviours (Diamond, 2013; Miller and Cohen, 2001). These higher-order cognitive functions involve *active* maintenance of goal presentations and the means to achieve these goals (Miller and Cohen, 2001). In this process, different types of information processing, different sensory modalities (e.g. visual and auditory), and different systems responsible for response execution, memory updating and retrieval, and emotional evaluation are involved. Accordingly, a wide range of functions and brain regions are involved in EFs. These higher-order cognitive functions are also required for adapting and regulating behaviour, mental and physical health, and cognitive, social, and psychological development (Diamond, 2013). Deficits of EFs or executive dysfunctions are commonly observed in patients with psychiatric and mental disorders (Elliott, 2003; Reimann et al., 2020). It is important to consider EFs as a meta-cognitive, supervisory, or controlling system rather than being tied to particular cognition domains (Ward, 2020). Nevertheless, EFs are commonly described in terms of specific types of information processing or cognitive functions.

Traditionally, the concept of EFs was closely related to the distinction between two types of information processing: automatic versus controlled processing (Shiffrin and Schneider, 1977). In this framework, EFs refer to those behaviours and processes that require intentional, online exert of control. Another popular model of EFs is to categorise them into separate modular-type processes and specific cognitive functions. There is a general agreement about three core EFs: response inhibition (e.g. inhibitory control), working memory, and cognitive flexibility (Miyake et al., 2000). Similar to this, early works attempted to describe EFs in terms of certain kinds of information processing associated with specific behavioural tasks. These processes can be summarised in (1) task-setting and problem-solving abilities, (2) response inhibition abilities, (3) task switching abilities, and (4) multitasking (Ward, 2020). In addition to these well-established accounts of EFs, results of neuroimaging studies suggest several organising principles of EFs. One of these organising principles is related to hemispheric differences of the neural substrates of EFs, which considers dissociated functional roles of the left and right hemispheres. In one such model, left lateral prefrontal cortex (PFC) is considered specialised for task-setting functions and the right lateral PFC is specialised for monitoring performance (Stuss and Alexander, 2000). Another proposed model organises the neural substrates of EFs anatomically from anterior to posterior parts of the brain. In one such model, a posterior to anterior gradient is considered for the lateral PFC with a differential functional specificity of the dorsal (linked to action planning) versus ventral (linked to language and objects) routes (Badre and D’Esposito, 2009). The updated version of this theory emphasizes on separate brain networks that interact via local and global hierarchical structure (Badre and Nee, 2018).

**Figure 1.**
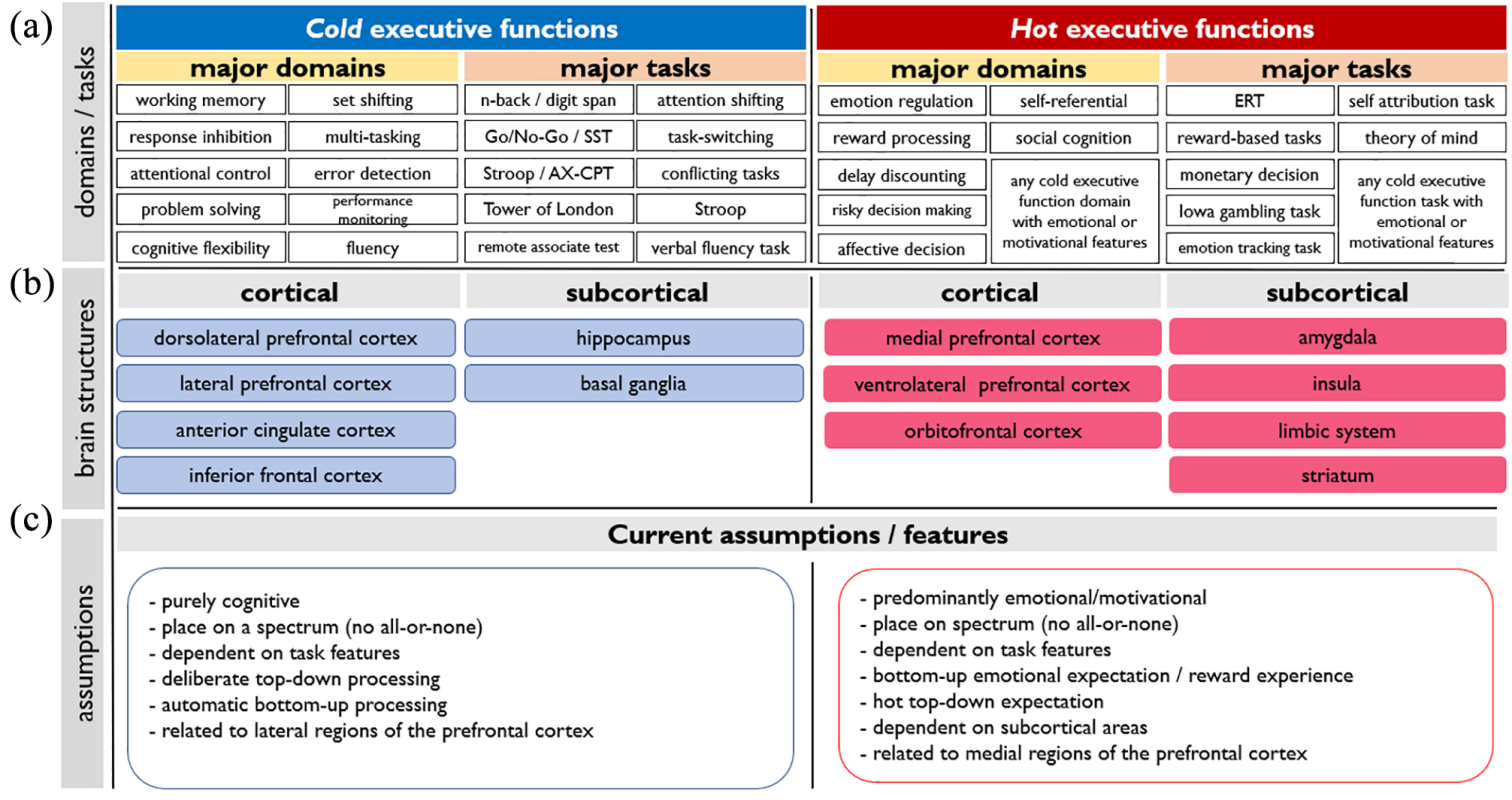
Current knowledge about domains and behavioural tasks of executive functions (a), involved brain structures (b) and underlying assumptions/features (c) of *hot* versus *cold* executive functions. SST: stop signal task; AX-CPT: AX Continuous Performance Task; ERT: emotional regulation task.

A recently emerging and perhaps the least controversial organising principle of EFs is to distinguish between EFs based on the extent they are related to emotion (e.g. *hot EFs*) or purely cognitive aspects (e.g. *cold EFs*) (Ward, 2020). *Hot* EFs, involve processing of information related to reward, emotion, and motivation, while *cold* EFs involve purely cognitive information processing. Examples of *hot* and *cold* EFs are ‘monetary delay discounting’ and ‘working memory letter’ tasks, respectively. The *hot* versus *cold* principle has several advantages for organising EFs. First, in this model both cognition and emotion are considered. Second, it presents EFs in a spectrum-like model, indicating that all domains of EFs can be *hot* or *cold* depending on contextual information, and third, broader regions of the brain are considered for EFs. A network approach, however, is needed to more accurately depicts functional organization of hot vs cold domains of EF. The current knowledge of neural substrates of *hot* versus *cold* EFs distinct between lateral (*cold*-related) and medial (*hot*-related) regions of the PFC. This, however, is not limited to the PFC regions and other cortical and subcortical areas appear to be involved as well. Major domains, tasks, and neural substrates of *hot* versus *cold* EFs based on the currently available studies are summarized in Figure 1.

Although the *hot–cold* organising principle of EFs has been most often linked to the PFC (as shown in Figure 1), here we attempt to broaden this principle to cingulate areas as well, due to their significant involvement in executive and cognitive control functions. It is, however, notable that EFs and especially *hot* EFs are closely related to subcortical areas involved in emotional processing, including the amygdala, insula, striatum (including putamen, caudate, and nucleus accumbens) hippocampus, and brainstem (Ardila, 2019; Heyder et al., 2004; Pessoa, 2009). As the scope of this review is focused on prefrontal and cingulate cortices, the specific contributions of these subcortical regions will not be covered in detail here, but will be mentioned where required. In the next section, we describe *hot* and *cold* EFs with a specific focus on majorly involved cortical regions, namely, the PFC and cingulate cortex. Before that, we briefly mention methods in cognitive neuroscience that provide important insights into the relevance of brain areas, and networks associated with EFs.

### Studying EFs: neuroimaging versus non-invasive brain stimulation methods

Recent advances in the cognitive neurosciences have provided us with novel, non-invasive methods for studying human cognition. Neuroimaging, particularly functional magnetic resonance imaging (fMRI), has become a dominant tool in cognitive neuroscience research and especially human cognition (Dolan, 2008). The emergence of this method has revolutionized study of the living human brain and fMRI is the most widely used technique in cognitive neuroscience (Newman, 2019). fMRI relies on blood oxygenation level–dependent (BOLD) contrast, which arises due to the magnetic susceptibility of deoxyhaemoglobin (deoxy-Hb). To put it briefly, an increase of neural activity leads to an increase of blood volume and thus the proportion of oxygenated haemoglobin (oxy-Hb) in the region, resulting in an increased BOLD signal. This BOLD signal is indicative of brain activity. When it is time-locked to an event/stimulus, it can be used to reveal neural correlates of cognition. A region with enhanced activity refers to a local increase of brain metabolism during performance of an experimental task compared to the baseline. With fMRI, we can investigate which brain regions are activated during cognitive task performance, including EFs. Most of our knowledge about the brain regions involved in EFs comes from neuroimaging studies (Elliott, 2003; Yuan and Raz, 2014). However, they come with some limitations. Apart from a relatively poor temporal resolution, which is, however, not the case for electroencephalogram (EEG), the other well-known neuroimaging method, fMRI delivers correlational information about the involvement of brain areas and networks in human cognition. In other words, the evidence provided by brain imaging methods is purely *correlative* and does not allow us to infer *causal* relationships between brain and behaviour.

While such correlative information about brain–behaviour relations is valuable and informative, it does not allow to easily infer causality of brain–behaviour relationships. Here, tools that allow active manipulation of brain activity come into play. Non-invasive brain stimulation (NIBS) is a group of methods for modulating neural processes of the brain, enabling us to directly study how an experimentally altered neural activity affects behaviour (Polania et al., 2018). Transcranial magnetic stimulation (TMS) and transcranial electrical stimulation (tES) are two commonly used and well-established NIBS techniques. TMS is based on principles of electromagnetism which ultimately leads to electrical stimulation of brain regions in a focal way, and transcranial direct current stimulation (tDCS), the most common used tES methods, uses a weak, painless electrical current applied to the scalp, thereby modulating brain excitability in a more non-focal way (Nitsche and Paulus, 2000). Depending on a specific frequency (for TMS) and stimulation polarity/intensity/duration (for tDCS), different TMS and tDCS protocols can result in excitatory or inhibitory after-effects that might last for several minutes and in this case are linked to long-term potentiation or long-term depression (Polania et al., 2018). Due to such effects on cortical excitability and neuroplasticity, which are physiological foundations of cognition, these techniques have great potential for experimental investigation of the physiological foundations behind human cognition.

As briefly mentioned, various cortical and subcortical regions are involved in EFs. While neuroimaging methods can show the functional and structural correlates of EFs in the brain, with NIBS (e.g. TMS and tDCS) we can further complement our knowledge of the brain regions/networks supporting EFs. In this review, we will mostly focus on the evidence coming from these methods (i.e. fMRI, TMS, and tDCS) in order to picture how *hot* versus *cold* EFs are organised in the brain. We focus mainly on studies conducted in healthy individuals in this review. However, due to high relevance of *hot* versus *cold* cognition in neuropsychiatric disorders, we discuss important clinical implications of this distinction at the end. A brief description of the research methods used in the studies of this review is summarised in Table 1.

**Table 1.** Characteristics of commonly applied neuroimaging and non-invasive brain stimulation methods for studying human cognition.

Method		Type	Delivered information	Invasiveness	Principle of action	Resolution/focality
Neuroimaging	fMRI	Recording	Correlative	Non-invasive	Brain haemodynamic	High spatial
						Low temporal
	EEG	Recording	Correlative	Non-invasive	Brain electrical activity	Low spatial
						High temporal
Non-invasive brain stimulation	TMS	Stimulation	Causal	Non-invasive	Electromagnetic stimulation	Focal
	tES (e.g. tDCS)	Stimulation	Causal	Non-invasive	Electrical stimulation	Non-focal

fMRI: functional magnetic resonance imaging; EEG: electroencephalogram; TMS: transcranial magnetic stimulation; tES: transcranial electrical stimulation.

## Hot versus cold EFs in the PFC

In traditional and contemporary conceptualisations of EFs, there is a consensus that the frontal lobe and especially the PFC have a critical role (Miller and Cohen, 2001). The PFC has extensive connections with almost all sensory systems, cortical regions, and subcortical structures involved in action, motor response, memory, emotion, and affect (Miller and Cohen, 2001). Our focus here is on how PFC structures are related to *hot* versus *cold* EFs. Broadly speaking, the most basic anatomical division within the PFC defines three cortical areas: the lateral PFC, the medial PFC, and the orbital PFC. The lateral PFC lies anterior to the premotor areas and the frontal eye fields and is situated close to the surface of the skull. It includes the dorsolateral prefrontal cortex (DLPFC) (Brodmann’s areas 46 and 9) and the ventrolateral prefrontal cortex (VLPFC) (Brodmann’s areas 44, 45, and 47) (Ward, 2020). The medial PFC lies between the two hemispheres and anterior to the corpus callosum and the anterior cingulate cortex (ACC) (Brodmann’s area 24 and adjacent regions). The orbitofrontal cortex (OFC) lies above the orbits of the eyes and the nasal cavity (Brodmann’s areas 11, 12, 13, and 14). It is of note that the OFC is functionally and anatomically related to the ventral part of the medial PFC and is sometimes referred to as the ventromedial prefrontal cortex (VMPFC) (Brodmann’s area 10, 14, 25, and 32 and parts of 11, 12, and 13) (Öngür and Price, 2000), but these areas are not identical at finer anatomical divisions (Ward, 2020).

The EF domains related to these areas can be classified in different ways. One popular classification is to functionally specify these areas based on the extent to which these are involved in *hot* (e.g. emotion and motivation-related) and/or *cold* EFs (e.g. purely cognitive). *Hot* EFs mainly involve the orbital and medial PFC, including the OFC and VMPFC, and *cold* EFs engage the lateral PFC, including the DLPFC and VLPFC (Öngür and Price, 2000; Stuss, 2011; Ward, 2020). Functionally speaking, *hot* EFs are top-down cognitive processes that operate in contexts with significant emotional and motivational salience, gratification, rewards and/or punishment (Zelazo and Carlson, 2012; Zelazo et al., 2005). Examples of *hot* EF are delay discounting, affective/risky decision-making, and interpersonal and social behaviour. *Cold* EFs are top-down cognitive processes that are logically based or mechanistic (Chan et al., 2008) and operate in affectively neutral contexts (Zelazo and Carlson, 2012). Examples of *cold* EFs include working memory, response inhibition, attentional control, and planning as far as these functions are not presented in an emotional context. In what follows, we provide evidence from neuroimaging (i.e. fMRI) and brain stimulation studies (i.e. TMS and tDCS) about the relation of *hot versus cold* EFs to different PFC areas.

### Neuroimaging studies

#### PFC and cold EFs

A large body of evidence from neuroimaging studies show that the lateral PFC, including DLPFC and VLPFC, are involved in *cold* EFs. Response inhibition, the ability to suppress unrelated or inappropriate stimuli/responses, is a core *cold* component of EFs. It is well-established that a specific region of the PFC, the right inferior frontal gyrus (r-IFG), is critical for inhibitory control (Aron et al., 2004, 2014; Hampshire et al., 2010). The r-IFG is moreover connected with the ACC, involved in error detection, and the lateral OFC when conveying information from non-reward systems (Du et al., 2020). The left IFG is also involved in verbal fluency, another major *cold* EF domain (Costafreda et al., 2006). The lateral PFC, including the DLPFC, is another well-documented region actively involved in working memory updating and tasks requiring executive control (Lemire-Rodger et al., 2019; Wagner et al., 2001). The PFC, however, should be considered as a part of a larger brain network, the fronto–cingulo–parietal network, that supports cognitive control via interaction of different cortical (and also subcortical) structures.

**Figure 2.**
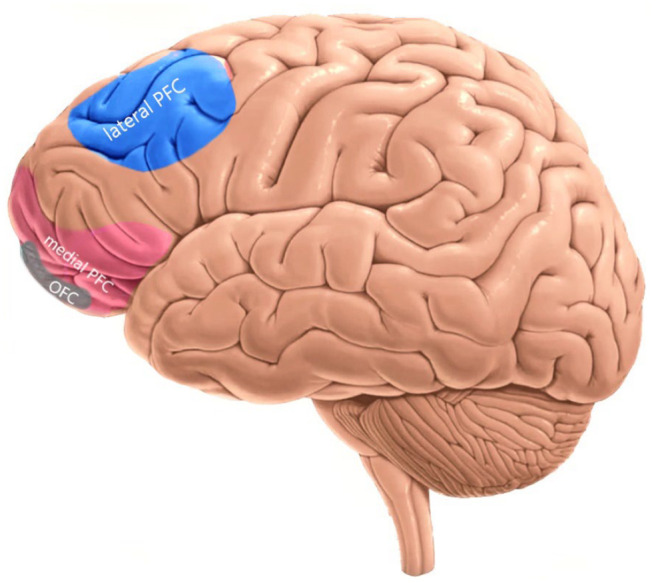
Lateral view of the prefrontal cortex (PFC) regions and association with *hot* and *cold* EFs. The lateral PFC includes dorsolateral prefrontal cortex (DLPFC) and ventrolateral prefrontal cortex (VLPFC) that are predominately involved in *cold* EFs (in blue). The medial PFC and orbitofrontal cortex (OFC) are predominantly involved in *hot* EFs. The *hot* PFC regions have extensive connections with several subcortical structures that process emotion and motivation will be discussed later (Figure 4). Marked regions are close approximate to the intended regions. Also note that circuit nodes and connections are excluded in this and later figures for clarity.

A great example of a *cold* EF and its association with subregions of PFC is navigation behavior. Planning, decision-making, goal-coding, and adaptive behavior are those domains of EFs required for real-world navigation (Patai and Spiers, 2021), all of which are functionally *cold*. At anatomical level, navigation behavior involves interaction of subregions of PFC (e.g., DLPFC, VLPFC), cingulate cortex (dorsal ACC) as well subcortical regions such as hippocampus (Patai and Spiers, 2021). An fMRI meta-analytic study of 193 studies revealed a common pattern of activation in the lateral PFC, dorsal ACC, and parietal cortex across major *cold* EF domains (working memory, inhibition, flexibility, and planning) (Niendam et al., 2012), indicating that *cold* EFs are supported by this cognitive control network with the DLPFC as a key region (Figure 2). The connectivity between the lateral PFC and dorsal ACC indicates that these regions are rather involved in *cold* EFs which are discussed in more detail in the section dedicated to the cingulate cortex.

#### PFC and hot EFs

A large and compelling body of evidence from neuroimaging studies shows that the medial and orbital PFC, specifically the VMPFC and OFC, are involved in cognitive functions related to reward, emotion, motivation, and social evaluation. During cognitive control of emotional stimuli, the medial PFC and OFC are usually activated (Ochsner and Gross, 2005). These regions, however, interact with the lateral PFC (e.g. VLPFC) and ACC during effortful control mechanisms when it comes to emotional and motivational stimuli (Ochsner and Gross, 2005; Pessoa, 2009). This indicates that *hot* EFs involve both brain regions involved in cold executive control (e.g. lateral PFC and ACC), and those involved in processing of emotion and motivation. One major *hot* EF is risky decision-making or decision-making under uncertainty. Neuroimaging studies have repeatedly shown that the VMPFC and OFC are involved in decision-making under uncertainty (Bechara et al., 2000; Clark et al., 2008; Fellows and Farah, 2007; Lipsman et al., 2014; Windmann et al., 2006). Delay discounting or temporal discounting is another classic example of *hot* EFs. Here again, studies show a prominent involvement of the VMPFC and OFC (Wang et al., 2016). What makes the medial–orbital PFC at least partially relevant for emotional and motivational processing is their connectivity with subcortical structures such as the limbic system, amygdala, and insula (Matyi and Spielberg, 2020; Sharpe and Schoenbaum, 2016). These regions are also connected with the posterior cingulate cortex (PCC), the counterpart region in the cingulate cortex which is discussed in the next section. Indeed, proposed models for delay-discounting behaviour in humans based on neuroimaging data assume that a unitary system encompassing the medial PFC, including VMPFC, and PCC are involved in immediate and delayed reward evaluation (Kable and Glimcher, 2007; Peters and Büchel, 2010). In this line, an fMRI study showed coactivation of the VMPFC and PCC during monetary reward encoding (Lin et al., 2011). Another fMRI study showed that when people consider themselves to experience a positive future, greater activity was observed in the VMPFC and PCC, indicating the connecting of these regions as well (Blair et al., 2013).

### NIBS studies

Attentional control is a core component of *cold* EFs. NIBS studies have shown that both TMS and tDCS over the left, right, or bilateral DLPFC enhance selective attention (Gladwin et al., 2012; Pecchinenda et al., 2015; Vanderhasselt et al., 2010). Other NIBS studies have moreover shown a performance-enhancing effect of increasing activity of the lateral PFC, including DLPFC and r-IFG, on attentional control and sustained attention (Coffman et al., 2012; Hwang et al., 2010). Regarding inhibitory control, the DLPFC and the IFG, with a right hemispheric predominance, are involved in response inhibition by both tDCS and TMS studies (for a review, see Brevet-Aeby et al., 2016). Working memory is another major component of *cold* EFs which was widely studied by NIBS. Recent review and meta-analytic studies have confirmed an enhancing effect of increased activity of DLPFC on working memory task performance (Bagherzadeh et al., 2016; Brunoni and Vanderhasselt, 2014; Hill et al., 2016; Mancuso et al., 2016). Another recent relevant meta-analysis investigated the effects of prefrontal tDCS here on executive function and found that anodal tDCS over the DLPFC increases performance of updating tasks and global EF performance under specific stimulation parameters (Imburgio and Orr, 2018). A recent tDCS study that targeted the DLPFC, temporal cortex, and posterior parietal cortex showed that DLPFC activation contributes to EFs regardless of task modality (semantic, phonemic, and visuospatial) (Ghanavati et al., 2019). Other studies show also enhanced problem-solving, and cognitive flexibility as a result of increased activity of lateral PFC regions via NIBS (Cerruti and Schlaug, 2009; Lucchiari et al., 2018; Metuki et al., 2012; Nejati et al., 2018a). These studies clearly show that the DLPFC and lateral PFC regions are involved in working memory and other *cold* EFs, although these structures are involved in specific aspects of emotional processing too (Nejati et al., 2021; Lindquist et al., 2012).

Regarding *hot* EFs, numerous NIBS studies show involvement of medial and orbital PFC regions. The involvement of the medial–orbital PFC (e.g. VMPFC and OFC) in reward and emotion processing is well-documented by both tDCS (Abend et al., 2019; Manuel et al., 2019) and TMS studies (Konikkou et al., 2017). A recent tDCS–fMRI study showed a causal link between VMPFC activation and the experience and regulation of anger, a *hot* EF domain, in an anger-provoking game involving fair and unfair offers, supporting its role in anger regulation (Gilam et al., 2018). Activity of the VMPFC in this study was coupled with both ACC and PCC activation, depending on the specific offer with more PCC activation during unpleasant offers. Social variables that include evaluation, interaction, theory of mind, and empathy are also considered *hot* EFs and NIBS studies have shown that activation of the VMPFC modulates such social variables (Adenzato et al., 2017; Chib et al., 2013; Li et al., 2020; Salehinejad et al., 2020a). A recent tDCS study specifically investigated the interaction of the DLPFC and OFC in *hot* versus *cold* EFs by applying excitability-enhancing anodal and excitability-reducing cathodal stimulation (Nejati et al., 2018a). Participants conducted response inhibition and problem-solving tasks as measures of *cold* EFs and risky decision-making and delay-discounting tasks as measures of *hot* EFs while receiving combined left DLPFC-right OFC stimulation. Increased activity of the left DLPFC concurrent with decreased activity of the OFC prominently improved *cold* EFs while *hot* EFs were enhanced under both protocols, those that activated the left DLPFC and the right OFC. The results of this study suggest that *hot* and *cold* EFs are placed on a spectrum, with lateral and medial–orbital contributions to *cold* and *hot* EFs, respectively, and that no EFs are purely *cold* or *hot.* This depends to the extent that each EF domain involves emotion/reward or cognition processing which determines engagement of relevant brain region. The brain regions should be predominantly, but not purely, considered *cold* and *hot* as well and this is determined by task feature too.

## Hot versus cold EFs in the cingulate cortex

The major anatomical divisions in the cingulate cortex include the anterior, mid, and posterior cingulate cortices, named ACC, MCC and PCC, respectively (Caruana et al., 2018; Vogt, 2005) although some classifications only include ACC and PCC. Here our focus is specifically on the ACC and PCC. Studying involvement of the cingulate cortex in EFs has been mostly limited to the anterior portion of the cingulate cortex or ACC (Brodmann’s area 24 and adjacent regions). The ACC is traditionally linked to the ability of error detection, a cognitive mechanism that monitors for errors and recalibrates task performance accordingly (Carter et al., 1998). As mentioned earlier, one fundamental domain of EFs is inhibitory control or response inhibition (Miyake et al., 2000) which is usually involved in situations with conflicting stimuli. The Stroop test and Go/No-Go tasks are well introduced behavioural examples with conflicting and competing stimuli. Conflict monitoring signals the need for increased cognitive control to resolve current conflicts, and here, the ACC can be linked to EFs (Cole et al., 2009; Shenhav et al., 2013). The involvement of the ACC in error detection abilities, which requires attentional control, suggests its predominant role in *cold* EFs. However, there is compelling evidence for an involvement of the ACC in emotion and reward–related processes as well. In fact, the ACC can be subdivided into areas differentially related to cognitive versus emotional functions. The dorsal ACC is linked to cognitive, whereas the ventral ACC is linked to emotional processing (Gasquoine, 2013; Lockwood and Wittmann, 2018). In addition to the ventral ACC, the PCC has been increasingly studied in recent years and linked to some domains of EFs related to *hot* cognition (Platt and Plassmann, 2014). In what follows, we present evidence from fMRI and brain stimulation studies about how *hot* and *cold* EFs are linked to the cingulate cortex with a primary focus on the ACC and PCC.

### Neuroimaging studies

#### ACC and cold/hot EFs

The relation of the ACC to *cold* EFs is based on its primary role in conflict detection during information processing, which signals the need to engage *top-down* attentional control and performance monitoring (Petersen and Posner, 2012). In this line, an early fMRI study aimed to investigate which levels of processing are being monitored by the ACC during performance of a task with conflicting stimuli and responses. It was shown that the ACC has a highly specific contribution to EFs through detection of conflicts at response level which usually occurs late during information processing (van Veen et al., 2001). This suggests that the executive control exerted by ACC is different from the contribution of the DLPFC. This was confirmed in another fMRI study that specifically compared the roles of the DLPFC versus ACC in attentional control. Attentional control is a clear example of *cold* EFs as it requires exerting control over the goal, monitoring of the goal, and the processes needed to achieve the goal. It is a fundamental component of executive control that comes into play in almost all EF domains and is traditionally linked to the DLPFC (Miller and Cohen, 2001). In that fMRI study, it was, however, shown that not only the DLPFC takes a leading role in implementing top-down attentional control, but also that the ACC is involved in specific additional aspects of attentional control, such as response-related processes (Milham et al., 2003). Regardless of the type of attentional control, the contribution of the ACC to this effortful process indicates that it is relevantly involved in *cold* EFs.

Involvement of the ACC in *cold* EFs is more precisely linked to the dorsal ACC. This region is a key hub in a network of brain regions involved in domain-general EFs in humans (Petersen and Posner, 2012; Shenhav et al., 2013); however, this ‘domain-general’ region has also a domain-specific function related to stimuli valence. An fMRI study in healthy participants showed that while dorsal ACC activity is required for processing task-irrelevant information during the Stroop task performance, which is distracting due to its *cognitive* content, the ventral ACC is activated during presentation of task-irrelevant information which is distracting due to *emotional* content (Mohanty et al., 2007). This study is a good example of how cognitive versus emotional information in the context of conflicting stimuli is processed by dorsal versus ventral ACC, respectively. In other words, both dorsal and ventral ACC seem to be involved in effortful control over stimuli, but these areas differ with respect to the kind of stimuli processed, that is, cognitive or emotional. Another fMRI study in healthy subjects, as well as in patients with ACC lesions, found that the *dorsal* ACC is actively involved in *effortful* cognitive and motor behaviour in healthy individuals, but that these activities were blunted in patients with focal lesions of the ACC (Critchley et al., 2003). Together, these studies suggest that the dorsal ACC is involved in *cold* EFs. Another influential account, however, links dorsal ACC functions with motivation and reward–based decision-making (Wallis and Kennerley, 2011). One of the most recent accounts for the role of the dorsal ACC integrates these two perspectives and suggests that that the dorsal ACC plays a central role in decisions about the allocation of cognitive control based on a cost/benefit analysis that identifies the highest expected value of control (Shenhav et al., 2016). According to this theory, exerting effortful control (*cold* EF) is based on the analysed value of control (*hot* EFs), and the dorsal ACC has a central role in these processes.

**Figure 3.**
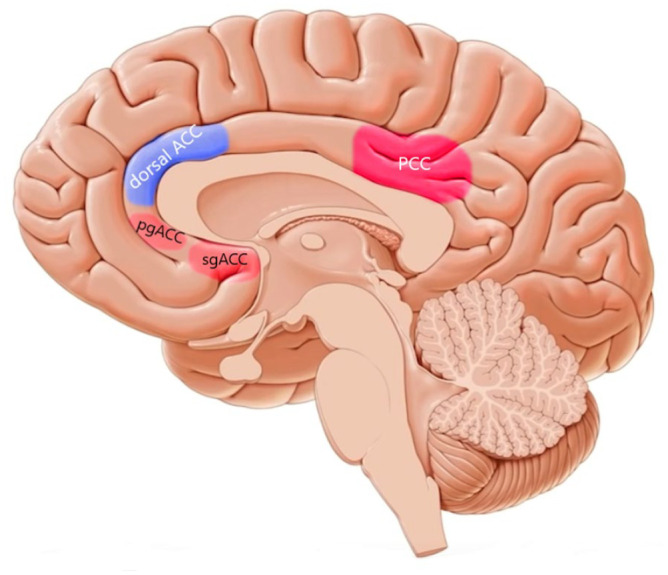
The cingulate cortex in the human brain and association with *hot* and *cold* EFs. The anterior cingulate cortex (ACC) includes dorsal ACC (dACC) that is predominately involved in *cold* (in blue) and ventral ACC, consisting of perigenual (pgACC) and subgenual (sgACC) that are predominately involved in *hot* EFs (in red), respectively. The posterior cingulate cortex (PCC) is predominantly involved in *hot* EFs (in red). Note that the anatomical borders of the cingulate cortex in this figure is based on the anatomical studies (see Caruana et al., 2018 and Vogt, 2005 for details). In some studies, the mid-cingulate cortex is part of the dACC. Marked regions are close approximate to the intended regions. Also, note that most circuit nodes and connections (specially subcortical regions) are excluded for clarity.

In contrast to the controversy over the functions of the dorsal ACC, there is a general agreement that the ventral region of the ACC is rather linked to emotional, motivational, and social information processing (Gasquoine, 2013; Lockwood and Wittmann, 2018). An fMRI study that measured performance monitoring with a valence-based task showed that the ventral ACC along with the PCC is specifically sensitive to the valence of feedback (Mies et al., 2011), specifically the perigenual ACC and subgenual ACC. These are two subregions of the ventral ACC that are linked with *hot* EFs involving emotion, motivation, and social decision-making (Lockwood and Wittmann, 2018). In accordance, a recent fMRI study showed connectivity between both perigenual and subgenual ACC and OFC/VMPFC, which are involved in emotional and motivational processing (Du et al., 2020). This might partially explain why the ventral ACC is rather involved in *hot* EFs, as it is structurally connected and anatomically closer located to those regions of the PFC that are relevant for *hot* EFs (Figure 3).

#### PCC and hot EFs

In comparison to the ACC, a relatively limited number of studies explored functional organisation of the PCC in EFs. Previous fMRI studies mainly investigated PCC activation during memory processes, specifically episodic memory. However, functional imaging studies consistently found that emotionally salient stimuli activate the PCC (Maddock, 1999), and the involvement of PCC in episodic and autobiographical memory could be due to its role in the interaction between emotion and memory (Maddock et al., 2001). The involvement of the PCC in emotion, and thus *hot* EFs, is anatomically related to its connectivity and coactivation with the amygdala, insula, and OFC (Vogt et al., 2000). An fMRI study found that the PCC was activated bilaterally during both unpleasant and pleasant, as compared to neutral words in a memory task (Maddock et al., 2003). Recent studies have provided more convincing evidence for the involvement of the PCC in emotional stimuli processing. Le et al. (2019) investigated neural substrates underlying behavioural avoidance in alcohol drinkers using a valence-based Go/No-Go task. Their major finding was increased activity in the PCC during motivated avoidance and incentivised inhibition of action which was correlated with sensitivity to punishment. In another recent TMS–fMRI study, 1 Hz rTMS was applied to the medial PFC of healthy participants who immediately thereafter underwent fMRI while performing an emotional self-referential task (De Pisapia et al., 2019). Neuroimaging findings revealed that the PCC was the only region that was specifically activated by negative-valence stimuli and as a result of TMS (TMS–valence interaction). Another recent study showed elevated functional connectivity between the PCC and subgenual PFC (e.g. ventral–medial PFC) as a maker of rumination, in depressed individuals versus healthy controls (Benschop et al., 2021). Rumination is a cognitive risk factor resulting from deficient cognitive control over negative emotions and a maladaptive self-referential processing and thus related to *hot* EFs. Overall, findings of these studies indicate relevant connectivity between the medial PFC and PCC, which will be discussed later in our prefrontal-cingular network model for *hot* versus *cold* EFs (Figure 3).

### NIBS studies

In contrast to neuroimaging studies that provide correlates of brain–behaviour relations, NIBS methods allow to infer the causality of these associations. The feasibility of NIBS to modulate ACC and PCC physiology is, however, limited in part due to the anatomical depth of these regions. Regarding *cold* EFs, some NIBS studies investigated the impact of the ACC stimulation on EFs and support the contribution of this region to these EFs.

In a recent tDCS study, using a high-definition (HD) stimulation protocol (i.e. 4 × 1 electrode montage), anodal and cathodal tDCS were applied over the dorsal ACC during performance of a cognitive and emotional cognitive Stroop task (To et al., 2018). Anodal stimulation over the dorsal ACC enhanced performance on the cognitive incongruent stimuli of the task, which requires effortful attentional control, while cathodal stimulation over the same region enhanced performance on the block including emotional incongruent stimuli. Furthermore, anodal stimulation significantly increased beta frequency band activity, which is associated with attentional control. A recent tDCS–fMRI study applied anodal tDCS over the ACC and measured behavioural performance in a colour-word Stroop task, and resting-state fMRI after stimulation (Khan et al., 2020). While behavioural findings showed enhanced Stroop task performance as a result of improved cognitive control, neuroimaging findings showed a significant decrease of functional connectivity of the cognitive control network, including ACC, which is associated with less effortful information processing. In a TMS study that targeted the ACC during a counting Stroop task performance (a *cold* EF), excitatory 10 Hz rTMS (Hayward et al., 2004) over both the dorsal and ventral ACC abolished Stroop interference. Together, NIBS studies show that stimulation of the dorsal ACC is associated with enhanced *cold* executive control.

Not many NIBS studies are conducted to modulate the activity of the PCC to explore its impact on cognitive functions. The TMS–fMRI study conducted by De Pisapia et al. (2019), which found a TMS–valance interaction for the activation of the PCC after applying TMS over the medial PFC, is, however, a relavant example. In this study, the neural basis of emotional content in self-referential processing, a *hot* EF, was investigated by stimulating the medial PFC with 1 Hz TMS. Participants then conducted a valence-based self-referential task. Stimulating the medial PFC activated a network of regions including the PCC which was specifically sensitive to emotionally negative aspects of the stimuli. In another study, the right DLPFC was stimulated with inhibitory TMS, and delay-discounting task performance was monitored during positron emission tomography (PET) scan. The PCC, and especially the posterior parietal lobule, which is part of the PCC, were activated during this task (Cho et al., 2012). Together, NIBS studies available so far show that activation of the PCC is observed during performing *hot* EF tasks (Table 2).

**Table 2.** Summary of hot versus cold executive functions in the studies of this review based on the applied tasks, techniques, and involved regions.

COLD executive functions – prefrontal regions					
Study	Domain	Used task	Technique	Region	Major finding
Aron et al. (2014) ^a^	Inhibitory control	Stop signal, Go/No-Go	fMRI	r-IFG	Inhibition as a central component of executive control relies on activation of the r-IFG
Hampshire et al. (2010)	Inhibitory control	Stop signal	fMRI	r-IFG	r-IFG is recruited in detecting inhibition cues
Costafreda et al. (2006)	Verbal fluency	Phonemic fluency, semantic fluency	fMRI	l-IFG	Dorsal–ventral regions of l-IFG are recruited in phonologic and semantic fluency
Wagner et al. (2001)	Executive control	Executive control, working memory	fMRI	DLPFC, VLPFC	DLPFC relevant for monitoring working memory stimuli, VLPFC relevant for maintenance and monitoring of information
Le et al. (2019)	Executive control	Executive control, working memory, task switching	fMRI	DLPFC, medial PFC	DLPFC involved in working memory, inhibition engaged lateral and superior medial PFC, task switching engaged bilateral DLPFC
Niendam et al. (2012) ^b^	Working memory, flexibility, inhibition, planning	n-back, PASAT, AX-CPT; task switching, WCST; Go/No-Go, flanker task; tower maze	fMRI	DLPFC, dACC	Common pattern of activation observed in the DLPFC, anterior cingulate and parietal cortices across executive function domains. Unique subcortical regions such as basal ganglia and cerebellum are involved
Gladwin et al. (2012)	Selective attention	Sternberg task	tDCS	DLPFC	tDCS over DLPFC improved reaction time of probes involving distracter stimuli
Pecchinenda et al. (2015)	Selective attention	Face-word interference task	tDCS	DLPFC	Anodal left DLPFC tDCS did not improve selective attention, cathodal left DLPFC tDCS reduced interference
Vanderhasselt et al. (2010)	Attentional control	Reaction-time attention task	HF-rTMS	DLPFC	HF-rTMS of the left DLPFC improved performance on the primary task, but not for the distracters
Coffman et al. (2012)	Attentional control	Attention network task	tDCS	Inferior frontal cortex (F10)	Alerting, but not orienting or executive attention, was significantly higher after 2 mA anodal tDCS
Hwang et al. (2010)	Attentional control	Continuous performance test	HF-rTMS	DLPFC	Fewer commission errors during trials after rTMS of left DLPFC as compared with sham stimulation
Brunoni and Vanderhasselt (2014) ^a^	Working memory	n-back task	tDCS, rTMS	DLPFC	Active vs sham rTMS presented faster and more accurate responses. Active vs sham tDCS presented faster responses only
Bagherzadeh et al. (2016)	Working memory	Verbal digit span task, 2-back task	HF-rTMS	DLPFC	rTMS of left DLPFC enhanced working memory performance
Imburgio and Orr (2018) ^a^	Global executive function	Inhibition, set-shifting, updating tasks	tDCS	DLPFC	Significant effect of anodal unilateral tDCS on updating but not on inhibition or set-shifting tasks, importance of stimulation parameters (electrode size, location) for observed effects
Ghanavati et al. (2019)	Global executive function	Verbal fluency task, semantic fluency task	tDCS	DLPFC	DLPFC activation contributes to EFs regardless of task modality (semantic, phonemic, and visuospatial)
Metuki et al. (2012)	Problem-solving	Verbal insight problem task	tDCS	DLPFC	Left DLPFC executive control enhances semantic processing of verbal insight problems
Cerruti and Schlaug (2009)	Cognitive flexibility	Remote associates test	tDCS	DLPFC	Anodal left DLPFC stimulation improves verbal problem-solving task which is dependent on significant executive function capacity
Nejati et al. (2018a)	Problem-solving, response inhibition	Tower of Hanoi, Go/No-Go test	tDCS	DLPFC	Response inhibition and problem-solving were prominently affected by anodal l-DLPFC–cathodal OFC stimulation
COLD executive functions – cingulate cortex					
Petersen and Posner (2012) ^a^	Attentional control, performance monitoring	Attentional tasks	fMRI	ACC, dACC	Established role of the ACC in top-down control, conflict detection and performance monitoring
van Veen et al. (2001)	Conflict detection	Flanker interference task	fMRI	ACC	ACC is responsive to detection of response conflict
Milham et al. (2003)	Top-down attentional control	Stroop task	fMRI	ACC	ACC is involved in specific aspects of attentional control, such as response-related processes
COLD executive functions – cingulate cortex					
Study	Domain	Used task	Technique	Region	Major finding
Shenhav et al. (2013) ^a^	Cognitive control	Control-demanding tasks (e.g. Stroop)	fMRI	dACC	dACC is involved in allocation of control based on an evaluation of the expected value of control
Mohanty et al. (2007)	Cognitive–emotional control	Emotion-word Stroop, colour-word Stroop	fMRI	dACC, rACC	Differential engagement dACC and rACC in cognitive and emotional processing, respectively
Critchley et al. (2003)	Motor control	Effortful motor task	fMRI/lesion	dACC	dACC is involved during effortful cognitive and motor behaviour
To et al. (2018)	Cognitive–attentional control	Emotional cognitive Stroop task	HD-tDCS	dACC	Anodal and cathodal tDCS over dACC enhanced performance on the cognitive and emotional incongruent stimuli, respectively
Khan et al. (2020)	Cognitive–attentional control	Colour-word Stroop task	tDCS–fMRI	ACC	Anodal tDCS improved behavioural performance, significant decrease of functional connectivity of the cognitive control network including ACC
Hayward et al. (2004)	Cognitive–attentional control	Stroop task	HF-rTMS	ACC	HF-rTMS dorsal and ventral ACC abolished Stroop interference (performance enhancement)
HOT executive functions – prefrontal regions					
Ochsner and Gross (2005) ^a^	Cognitive control of emotion	N/A	fMRI	Medial PFC, OFC, ACC	Medial PFC, OFC, and ACC are involved in emotional appraisal systems; VLPFC, OFC, ACC, and medial–lateral PFC are involved in attentional control over emotions
Pessoa (2009) ^a^	Cognitive control of emotion and motivation	N/A	fMRI/PET	ACC, amygdala, nucleus accumbens	ACC is engaged in integrating affective signals in the amygdala and nucleus accumbens with control signals in the PFC
Clark et al. (2008)	Risky decision-making	Iowa gambling task	Lesion study	VMPFC	VMPFC damage was associated with riskier decision-making
Fellows and Farah (2007)	Value-based decision-making	Preference judgement task	Lesion study	VMPFC	VMPFC damage, but not frontal lobe damage leads to impaired decision-making under certainty
Lipsman et al. (2014)	Affective decision-making	Emotion tracking task	EEG	VMPFC	15–20 Hz coherent activity in VMPFC is a functional signature of a valuation process
Windmann et al. (2006)	Risky decision-making	Iowa gambling task	fMRI	OFC	Lateral OFC is involved in processing of unsteady (changing) rewards
Wang et al. (2016)	Risky decision-making	Delay-discounting and stop signal tasks	MRI/fMRI	VMPFC	Grey matter of middle frontal gyrus and connectivity between frontal pole and VMPFC predicted discounting rate but not impulsive choice
Kable and Glimcher (2007)	Risky decision-making	Monetary-discounting task	fMRI	Medial PFC, PCC, ventral striatum	Activity in the ventral striatum, medial PFC, and PCC tracks the subjective value of delayed monetary rewards
Peters and Büchel (2010)	Value-based decision-making	Monetary-discounting task	fMRI	PCC, VMPFC	Greater activity in PCC and right VMPFC in the monetary condition with subject-specific reward vs control (only monetary)
Lin et al. (2011)	Reward processing	Learning task with monetary or social rewards	fMRI	VMPFC, PCC	Coactivation of the VMPFC and PCC during monetary reward encoding
HOT executive functions – prefrontal regions					
Study	Domain	Used task	Technique	Region	Major finding
Blair et al. (2013)	Valenced bias estimation	Valenced bias estimation task	fMRI	VMPFC, PCC	Positive bias estimation was associated with greater activity within VMPFC and PCC
Manuel et al. (2019)	Emotional delay discounting	Valenced delay-discounting task	tDCS	VMPFC	Anodal VMPFC tDCS decreased impulsivity and cathodal tDCS increase impulsivity after positive emotions
Abend et al. (2019)	Emotion regulation	Emotion induction task	tDCS–fMRI	Medial PFC	Active tDCS reduced intensity of perceived negative emotions. sgACC activation correlated with reported emotion intensity
Gilam et al. (2018)	Emotion regulation	Anger-infused ultimatum game	tDCS–fMRI	VMPFC, PCC	Activity of the VMPFC was coupled with PCC activation during unpleasant offers
Li et al. (2020)	Social cognition, conformity	Social decision-making task	tDCS	VMPFC	Cathodal stimulation of VMPFC increased conformity tendency
Adenzato et al. (2017)	Theory of mind	Reading the Mind in the Eyes task, attribution of intentions task	tDCS	Medial PFC	Anodal tDCS over the medial PFC enhances ToM in females but not in males
Nejati et al. (2018a)	Risky decision-making	Risk-taking and delay-discounting tasks	tDCS	OFC, DLPFC	Activation of both left DLPFC and the right OFC with anodal tDCS improved performance on hot EFs tasks
Figner et al. (2010)	Risky decision-making	Monetary-discounting task	rTMS	Lateral PFC	Disruption of left lateral PFC, increased choices of immediate rewards over larger delayed rewards
HOT executive functions – cingulate cortex					
Lockwood and Wittmann (2018) ^a^	Social decision-making	Social cognition tasks	fMRI	Ventral ACC, subgenual ACC, perigenual ACC	Subgenual ACC and perigenual ACC are activated during social decision-making and social prediction error
Mies et al. (2011)	Valence-based time-estimation	Time-estimation task	fMRI	rACC, PCC	The rACC and PCC were primarily sensitive to the valence of the feedback and more active after positive feedback
Maddock et al. (2003)	Emotional memory	Valenced memory task	fMRI	PCC, subgenual ACC	The PCC was significantly activated bilaterally during both unpleasant/pleasant vs neutral words with strongest activity in subgenual ACC
Le et al. (2019)	Incentivised inhibition	Reward Go/No-Go task	fMRI	PCC	Increased activity in the PCC during motivated avoidance and incentivised inhibition
De Pisapia et al. (2019)	Self-referential processing	Emotional self-referential task	TMS–fMRI	PCC	PCC was the only region that was specifically activated by negative-valence stimuli and as a result of TMS
Cho et al. (2012)	Risky decision-making	Delay-discounting task	LF-rTMS/PET	PCC	The PCC, and especially the posterior parietal lobule, were activated during task performance

DLPFC: dorsolateral prefrontal cortex; VLPFC: ventrolateral prefrontal cortex; dACC: dorsal anterior cingulate cortex; sgACC: subgenual anterior cingulate cortex; vACC: ventral anterior cingulate cortex; rACC: rostral anterior cingulate cortex; OFC: orbitofrontal cortex; TBS: Theta Burst Transcranial Stimulation; HF-rTMS: high-frequency (excitatory) transcranial magnetic stimulation; LF-rTMS: low-frequency (inhibitory) transcranial magnetic stimulation; tDCS: transcranial direct current stimulation; PET: positron emission tomography; VMPFC: ventromedial prefrontal cortex; PASAT: Paced Auditory Serial Addition Test; AX-CPT: AX-Continuous Performance Test; WCST: Wisconsin Card Sorting Task; N/A: not applicable; r-IFG: right inferior frontal gyrus; l-IFG: left inferior frontal gyrus; ToM: theory of mind.

a Review articles based on neuroimaging or brain stimulation studies.

b Meta-analysis articles based on fMRI or NIBS studies.

The studies in this table include selective neuroimaging/NIBS studies.

## A prefrontal-cingular network model for *hot* versus *cold* EFs

**Figure 4.**
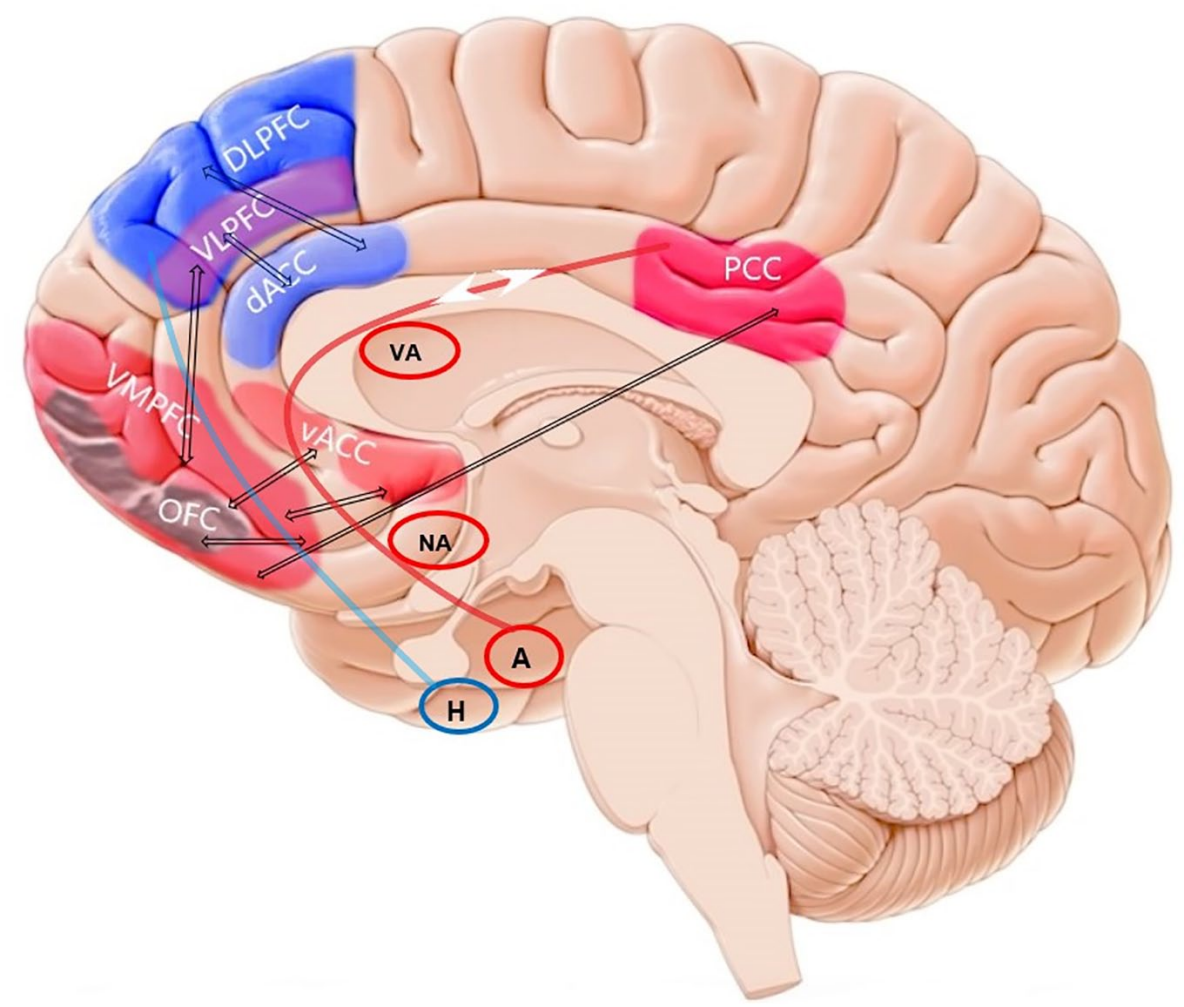
The *prefrontal-cingular network* in the human brain and association with *hot* and *cold* EFs. The lateral PFC, including DLPFC and VLPFC, along with dorsal ACC are predominantly related to *cold* EFs and can be considered as the *cold* stream. The PCC, medial and orbital PFC (VMPFC and OFC), and ventral ACC constitute the hot stream and are predominantly related to *hot* EFs. The VLPFC is also connected to medial and orbital PFC. The *hot* EFs stream is closely connected with several limbic structures that are involved in emotional and motivational processing (red curve). The connectivity between the hippocampus and lateral prefrontal cortex subregions is also relevant for major cold EFs such as working memory and navigation behavior. DLPFC: dorsolateral prefrontal cortex; VLPFC: ventrolateral prefrontal cortex; ACC: anterior cingulate cortex; dACC: dorsal anterior cingulate cortex; vACC: ventral anterior cingulate cortex; VMPFC: ventromedial prefrontal cortex; OFC: orbitofrontal cortex; PCC: posterior cingulate cortex; VA: ventral striatum; NA: nucleus accumbens; A: amygdala; H: hippocampus. Marked regions are close approximate to the intended regions. Note that some circuit nodes and connections specially with subcortical areas are excluded for clarity and that some connections (shown by arrows) may be indirect.

So far, we discussed how *hot* and *cold* EFs can be linked to different regions within the PFC and the cingulate cortex. Anatomical and functional connectivity between the cingulate and prefrontal cortices can explain functional organisations of *hot–cold* EFs in this network. Two major functional connectivity branches are considered in this proposed model: (1) the connectivity between the lateral PFC (e.g. DLPFC) and ACC (specifically the dorsal ACC) and (2) the connectivity between the orbital–medial PFC (e.g. VMPFC and OFC), ventral ACC, and PCC (Figure 4). In this section, we discuss a prefrontal-cingular network that may more comprehensively account for *hot* and *cold* EFs. As subcortical regions are highly involved in *hot* EFs, we also depicted major subcortical limbic structures involved in emotional and motivational processing which are connected to *hot*-related prefrontal-cingular structures.

The cingulate cortex in monkeys and humans has extensive connections with the PFC (Pandya et al., 1981). The dorsal ACC (Brodmann’s area 32) projects mostly to the lateral PFC, including DLPFC, and the mid-OFC (Pandya et al., 1981), and the ventral ACC (Brodmann’s area 25), which is part of the VMPFC, has connections with subcortical regions like amygdala and insula, and projects also to the VMPFC, OFC also lateral PFC. The PCC (Brodmann’s area 31), however, projects to the VMPFC, ventral ACC, and OFC (Leech and Sharp, 2014). Although there is a relative overlap between the ACC and PCC connectivity with PFC regions at the anatomical level, anterior and posterior parts of the cingulate cortex show a more differentiated functional specificity. As discussed in the previous section, both neuroimaging and brain stimulation studies show that the dorsal anterior part of the cingulate cortex is mainly involved in cognitive control functions (Critchley et al., 2003) such as response inhibition and conflict monitoring (Botvinick et al., 2004). From a functional perspective, these EF domains are mostly relevant for *cold* EFs and similar to those domains the lateral PFC is involved in, although a functional difference is observed for the timing of attentional control exerted by the DLPFC and dorsal ACC (Milham et al., 2003). The connectivity between the lateral PFC and ACC and their coactivation during cognitive control tasks supports this functional link (Tik et al., 2017). The ventral ACC is mainly involved in exerting control over emotional stimuli which indicates that the ACC is involved in both *cold* and *hot* EFs (Gasquoine, 2013; Lockwood and Wittmann, 2018). However, the cognitive functions specific to the posterior part of cingulate cortex (e.g. PCC) are shown to be involved in emotional processing, value-based decision-making, subjective valuation, and motivational states (Platt and Plassmann, 2014; Vogt et al., 2000). In this connection, neuroimaging studies have shown coactivations between the PCC and the medial–orbital PFC (e.g. VMPFC and OFC) (De Pisapia et al., 2019; Le et al., 2019; Lin et al., 2011; Vogt et al., 2000).

This relative functional specificity of the anterior versus posterior parts of the cingulate cortex seems similar to the ‘*hot*–*cold*’ organising principle of EFs in the PFC. In the PFC, the *hot* versus cold organisation is proposed based on functional differences between the lateral versus medial–orbital regions. Neuroimaging and brain stimulation studies have documented that *cold* EFs are rather supported by the lateral PFC, while *hot* EFs are related to the medial–orbital PFC (Nejati et al., 2018a; Ochsner and Gross, 2005; Öngür and Price, 2000; Peterson and Welsh, 2014; Ward, 2020). Integrating this functional differentiation in the PFC and cingulate cortex with respect to *hot* versus *cold* EF allows us to consider a broader network. According to the prefrontal-cingular network, the lateral PFC (e.g. DLPFC and VLPFC) is functionally more closely related to the dorsal ACC, while the medial–orbital PFC (e.g. VMPFC and OFC) is functionally and anatomically more closely related to the ventral ACC and PCC. This does not, however, exclude a contribution of the dorsal ACC, which is documented to be involved in emotion, affect, and pain, to *hot* EFs (Shackman et al., 2011). Considering a purely segregationist model for the ACC seems to be no longer appropriate (Shackman et al., 2011), however, our discussion here is limited to EFs and specifically *hot* versus *cold* domains, which seems to be functionally supported by different regions of the cingulate cortex.

It is important to consider the following additional aspects with regard to the proposed prefrontal-cingular network. First, the involvement of the lateral PFC and dorsal ACC in *cold* EFs, and the medial–orbital PFC, ventral ACC, and PCC in *hot* EFs does not imply that these regions are functionally limited to *cold* or *hot* cognition. They rather have a predominant functional specificity. As shown in previous studies, there is an interplay between these cold-related and hot-related regions (Le et al., 2019; Nejati et al., 2018a) and importantly these regions show coactivations depending on specific task features. Second, the *hot* versus *cold* EFs distinction should not likewise be considered as representing two separate and unrelated domains of EFs. Although some domains are most purely cognitive, such as inhibitory control or working memory, they might be enriched by emotional features depending on the task, stimuli, and the context used for measuring them. Finally, it should be taken into account that we narrowed our discussion to the prefrontal and cingulate cortices. The *prefrontal-cingular network* includes the cortical regions most closely involved in EFs based on previous studies. This is not meant to underestimate the engagement of other brain regions, especially subcortical limbic regions, the amygdala–hippocampal systems, and sensorimotor regions of the dorsal striatum (e.g. putamen and caudate nuclei) in EFs, which were, however, beyond the scope of this review.

## Clinical implications of hot–cold EFs for neuropsychiatric disorders

The *hot–cold* distinction of cognition has important clinical implications for both characterising and applying appropriate treatment of neuropsychiatric disorders. In the majority of neuropsychiatric disorders, the core pathophysiology involves cortico-subcortical regions of the brain, and here the prefrontal-cingular network is highly involved (Heilbronner and Haber, 2014; Miller and Cummings, 2017). This is in line with the network approach in cognitive neuroscience, which assumes that a dynamically changing pattern of activity over several brain regions is critical for cognitive processes (Ward, 2020). Disturbances of these networks – structural and functional – are related to symptoms and pathophysiology of neuropsychiatric disorders. Accordingly, it is possible to identify symptom-relevant brain networks, and their disturbances, based on connectivity mapping of the human connectome which is one of the major approaches in current biological psychiatry. Abnormalities of the prefrontal-cingular and prefrontal-limbic networks are largely involved in the pathophysiology, symptom expression, and course of the major neuropsychiatric disorders, including but not limited to depression, schizophrenia, anxiety disorder, substance use, and impulse control disorders, as well as major neurodevelopmental disorders (attention-deficit hyperactivity disorder (ADHD) and autism). In what follows, we briefly discuss the respective pathophysiology of some of these disorders and outline whether their cognitive profiles (i.e. *hot* vs *cold*) are fundamental (central) for or rather manifest (relevant expression) in the psychopathology of each disease. A summary of the specific *hot* versus *cold* profile of each disorder is shown in Table 3.

**Table 3.** Hot–cold cognitive profile in major neuropsychiatric disorders.

Disorder	*Cold* cognition profile			*Hot* cognition profile		
	*Cognitive profile*	*Impaired domain*	*Brain region*	*Cognitive profile*	*Impaired domain*	*Brain region*
Depression	Deficient cold cognition (fundamental^a^)	Cognitive control (central)	DLPFC	Deficient hot cognition (manifest^b^)	Emotion regulation (rumination)	sgACC
	Cold cognition turned hot	Memory (verbal, working)	ACC	Negative hot top-down expectation	Emotional bias/perception	Amygdala
		Attention (sustained)	Hippocampus	Deficient hot bottom-up processes	Reward/punishment processing	Striatum including nucleus accumbens
		Time-estimation			Valenced cold cognition (e.g. affective Go/No-Go)	
Schizophrenia	Deficient cold cognition (fundamental, manifest)	Cognitive control	Lateral PFC	Deficient hot cognition	Social cognition (mind reading)	OFC
		Memory (verbal, working)	ACC		Risky decision-making	Amygdala
		Attention	Thalamus		Emotion recognition	
		Reasoning	SensorimotorHippocampus		Emotional appraisal	
		Processing speed				
		Time-estimation				
Anxiety disorders	Deficient cold cognition	Inhibition	DLPFC	Deficient hot cognition (fundamental, manifest)	Emotion regulation	Medial PFC
	Deficient attentional control	Set-shifting	ACC		Reward/punishment processing	VMPFC/OFC
		Attention				amygdala
		Working memory				
Substance use	Deficient cold cognition (fundamental)	Executive control	DLPFC	Deficient hot cognition (fundamental, manifest)	Reward/punishment processing	VMPFC/OFC
		Task switching	ACC	Deficient hot bottom-up processes	Valenced cold cognition (e.g. cue-dependent attention)	PCC
		Response inhibition	Basal ganglia			Insula, amygdala
						Nucleus accumbens
ADHD	Deficient cold cognition (fundamental, manifest)	Response inhibition	Inferior PFC (e.g. r-IFG)	Deficient hot cognition in few domains	Valenced cold cognition (e.g. executive reward processing)	Ventral striatum
		Working memory	DLPFC		Emotion regulation	Medial PFC
		Sustained attention	Parieto-temporal		Delay discounting	OFC
			Basal ganglia			VMPFC
Autism	Deficient cold cognition	Working memory	DLPFC	Deficient hot cognition (fundamental, manifest)	Emotion recognition	VMPFC
		Response initiation	IFG		Social inference	Precuneus
		Planning			Delay discounting	PCC
		Cognitive flexibility			Affective decision-making	Amygdala/insula

DLPFC: dorsolateral prefrontal cortex; ACC: anterior cingulate cortex; sgACC: subgenual anterior cingulate cortex; OFC: orbitofrontal cortex; PCC: posterior cingulate cortex.

a Fundamental refers to the condition in which respective deficit or profile has influence over other domains/deficits.

b Manifest refers to the deficit or profile that is more commonly observed, but not fundamental or core deficits.

### Depression

Emotional dysregulation is the core phenotype in depression and in agreement with this, deficits of *hot* cognition are a common manifestation in depression. From a *hot–cold* perspective, however, dysfunctional *cold* EFs, especially cognitive control deficits, are central for the psychopathology of depression, in line with cognitive theories of depression (Gotlib and Joormann, 2010). In other words, *cold* cognition turns *hot* in depression (Roiser and Sahakian, 2013). Deficient *cold* EFs are observed in cognitive control, working memory, and attention (Nikolin et al., 2021; Salehinejad et al., 2017), while *hot* EF deficits involve those EF tasks (mainly cold) that utilise emotionally valenced stimuli, and reward and punishment processing (Roiser and Sahakian, 2013). In accordance, neuroimaging studies show abnormalities of frontal-limbic structures that account for both *cold* and *hot* cognitive deficits (Keren et al., 2018; Rive et al., 2013). A central functional abnormality of the left and right PFC is proposed in depression with a hypoactivated left and hyperactivated right DLPFC, supported by the results of neuroimaging studies (Grimm et al., 2008). Modulation of the activity of these regions is consequently one focus of NIBS treatment in depression (Chen et al., 2020; Razza et al., 2020; Rostami et al., 2017). Treatment approaches in depression should consider fundamental cold executive dysfunctions as the primary target more than before in line with the *hot*–*cold* pathology of the disease explained above.

#### Schizophrenia

In schizophrenia, deficient *cold* cognition has been more extensively studied with respect to the disease pathophysiology and symptoms (Sheffield et al., 2018). A deficient *cold* cognitive profile seems to be both fundamental to and manifest of the symptoms and underlying pathophysiology. This is also in agreement with the developmental aspect of schizophrenia, including onset in adolescents, where cold cognition deficits are central (James et al., 2016). A well-documented deficient network in schizophrenia that is involved in *cold* cognition is the thalamocortical circuitry, especially the thalamus-PFC pathway (Giraldo-Chica and Woodward, 2017) and prefrontal-hippocampal connectivity (Meyer-Lindenberg et al., 2005). Deficient cognitive control, processing speed, memory (verbal, working), and reasoning are commonly reported *cold* EF deficits in schizophrenia (Table 3). However, impaired *hot* EFs, including risky decision-making, theory of mind, and emotion recognition are also reported in schizophrenic patients, and associated with psychotic symptoms (MacKenzie et al., 2017; Ruiz-Castañeda et al., 2020). Regarding treatment approaches, therapeutic targeting of *cold* EF deficits aligns, however, best with the fundamentally involved *cold* cognitive profile and pathophysiological characteristics of the disease.

### Anxiety disorders

Anxiety disorders are traditionally linked to emotional and threat-related difficulties, and thus, *hot* cognition deficits (e.g. emotion regulation, threat perception, reward–punishment processing) are central for the pathology of these disorders. These emotional difficulties are, however, strongly linked to deficits in several *cold* EFs that are stable over time (Nejati et al., 2018b; Zainal and Newman, 2018). Two well-known theories in this respect are the ‘Attentional Control Theory’ (Eysenck and Derakshan, 2011) and the ‘Cognitive Model’ of pathological worry (Hirsch and Mathews, 2012). In these theories, impaired *cold* EFs (specifically inhibition and set-shifting abilities), on one hand, and threat-related perceptual and attentional bias, on the other hand, are proposed to be responsible for the overwhelming experience of worry and anxiety. This effect is, however, dependent on the extent to which respective EF tasks include threatening stimuli or significant cognitive load (Leonard and Abramovitch, 2018). Neuroimaging and brain stimulation studies have shown functional and structural abnormalities of cortical regions related to both *hot* and *cold* EFs, with a specific focus on the prefrontal-amygdala network in anxiety disorders (Ironside et al., 2019). Here, a hyperactivation of the medial PFC (VMPFC and OFC) and ventral ACC (*hot* pathway), which is highly relevant for fear memory and extinction (Marcovic et al., 2021), and a hypoactivation of the DLPFC and dorsal ACC (*cold* pathway) are shown to be linked to hypersensitivity of the amygdala and other limbic structures (Vicario et al., 2019).

### Substance use disorders

A common and core feature of substance use disorder (SUD) is impaired control over craving, or impulsive behaviour. In accordance, here again deficits of both *cold* and *hot* EFs are central to the psychopathology of SUD. According to the neurocognitive model of addiction, *hot* and *cold* executive deficits play a fundamental and manifest role in different stages of addiction. In the first two stages (binge/intoxication and withdrawal/negative affect), a deficient reward system is central (Koob and Volkow, 2016), which is related to a deficient *hot* cognition, and in the preoccupation/anticipation stage, where craving behaviour dominates, a deficient executive control system is relevantly involved (Koob and Volkow, 2016), which is related to *cold* EFs. Therefore, both *hot* and *cold* EFs deficits are involved in the psychopathology of SUD, although *hot* manifestations of symptoms are predominant. The dual-process model of addiction similarly emphasises on both a *cold*-related ‘controlled’ system (related to the lateral PFC) and a *hot*-related ‘impulsive’ system (including mesolimbic and nigrostriatal pathways) (Wise, 2009). Novel treatment approaches in SUD have shown the relevance of targeting the *cold*-related ‘controlled’ as well as the *hot*-related ‘impulsive’ system by modulating activity of brain structures including DLPFC and ACC which are connected to reward system (Alizadehgoradel et al., 2020; Zhao et al., 2021).

### ADHD

ADHD is a major neurodevelopmental disorder, and executive dysfunctions are central for its psychopathology (Willcutt et al., 2005). Results of functional and structural neuroimaging studies, and behavioural studies exploring EFs show predominantly *cold* EF deficits in the psychopathology, and pathophysiology of ADHD (Antonini et al., 2015; Hobson et al., 2011; Molavi et al., 2020b; Rubia, 2018). Regarding *hot* EFs, results are mixed, with some studies reporting deficits in affective/motivational EF tasks (Nejati et al., 2020), while others report unimpaired *hot* EF functions (Antonini et al., 2015). However, neuroimaging, brain stimulation, and behavioural studies have recently shown an impairment of several *hot*-related cognitive processes and an involvement of medial PFC regions in *hot* EF task performance (Nejati et al., 2020; Rubia, 2018). It might be speculated that *hot* EF deficits in ADHD are caused by central *cold* executive deficits and do not exist independently (Van Cauwenberge et al., 2015). In accordance, the pathology of the functional activity profile of the brain in ADHD is more closely aligned with predominantly *cold* EF deficits with a fundamental involvement of the frontoparietal network (including IFG, DLPFC, ACC, and temporoparietal regions), the basal ganglia, and the cerebellum (Rubia, 2011). NIBS studies, in this line, have been mostly focused on improving *cold* EFs in ADHD (Salehinejad et al., 2019, 2020b).

### Autism spectrum disorder

Core symptoms in autism spectrum disorder (ASD) include deficits in reciprocity behaviours (required for successful social interaction), and repetitive behaviours. However, ASD is rather known as a disorder of social abilities, although this depends also on the phenotype of the disease. In contrast to this prevailing view, the majority of studies about EFs in ASD investigated *cold* EFs. Recently, however, *hot* EFs are studied more extensively in ASD. Briefly, these studies show that ASD involves deficits of both *cold* and *hot* EFs (Kouklari et al., 2018; Zimmerman et al., 2016). However, deficits related to *hot* cognition (e.g. reciprocity abilities and theory of mind) seem to be predominant (Kouklari et al., 2017, 2018; Zimmerman et al., 2016), which aligns with the central role of the medial PFC and PCC in the pathophysiology of ASD (Li et al., 2017; Patriquin et al., 2016). *Cold* EFs are also relevantly impaired, but these deficits might be secondary and largely restricted to those *cold* domains needed for the performance of *hot* EFs (Zimmerman et al., 2016). This is in line with the development of EFs in ASD. Cold but not hot EFs improve significantly as a function of age (Kouklari et al., 2018), suggesting that hot deficits are more fundamental for the psychopathology of ASD. Considering the heterogeneity of the disease, more detailed research is required to determine the *hot*–*cold* profile in ASD, and its subtypes.

### Other relevant disorders

*Hot–cold* executive dysfunctions and respective pathophysiology in the prefrontal-cingular network are prominent in psychopathology other neuropsychiatric disorders as well including but not limited obsessive–compulsive disorders (*cold*-deficits driven), borderline personality disorder (*hot*-deficits driven), and impulse control disorders (*cold–hot deficits* driven) (Gruner and Pittenger, 2017; Schulze et al., 2019). In this line, recent NIBS studies have also shown than modulating activity of the prefrontal-cingular and relevant subcortical regions are promising for the treatment of these disorders (Brevet-Aeby et al., 2016; Molavi et al., 2020a; Rostami et al., 2020).

## Conclusion

This review was focused on how *hot* and *cold* EFs can be functionally organised in the prefrontal and cingulate cortices. Based on evidence from neuroimaging and NIBS studies, we propose a *prefrontal-cingular network* that can explain neuronal correlates of *hot* versus *cold* EFs more comprehensively and in line with the current network-driven approach. In this network, the lateral PFC and associated regions (e.g. DLPFC, VLPFC, and IFG) along with the ACC, specifically the dorsal ACC, are more closely involved in *cold* EFs (e.g. attentional control, inhibition, error detection, and working memory), whereas the medial and orbital PFC regions (e.g. VMPFC and OFC) and ventral ACC along with the PCC are more relevant for *hot* EFs that involve emotional, motivational, reward/punishment based, and social stimuli. The extent to which these regions are *hot* or *cold* EF-related does not exclude a role of these networks in the other EFs, but rather indicates a gradual dominance for the respective type of information processing. The *hot–cold* distinction in EFs, and broadly in cognition, provide a novel, network-based approach for studying underlying pathophysiology in major neuropsychiatric disorders that usually come with both cognitive and emotional disturbances. This can promote more effective therapeutic intervention congruent with cognitive profile of the diseases.
